# Mesenchymal stem cells are short-lived and do not migrate beyond the lungs after intravenous infusion

**DOI:** 10.3389/fimmu.2012.00297

**Published:** 2012-09-26

**Authors:** E. Eggenhofer, V. Benseler, A. Kroemer, F. C. Popp, E. K. Geissler, H. J. Schlitt, C. C. Baan, M. H. Dahlke, M. J. Hoogduijn

**Affiliations:** ^1^Department of Surgery, University Medical Center RegensburgRegensburg, Germany; ^2^Transplantation Laboratory, Department of Internal Medicine, Erasmus Medical CenterRotterdam, Netherlands

**Keywords:** mesenchymal stem cell, infusion, localization, survival, lung, liver

## Abstract

Mesenchymal stem cells (MSC) are under investigation as a therapy for a variety of disorders. Although animal models show long term regenerative and immunomodulatory effects of MSC, the fate of MSC after infusion remains to be elucidated. In the present study the localization and viability of MSC was examined by isolation and re-culture of intravenously infused MSC. C57BL/6 MSC (500,000) constitutively expressing DsRed-fluorescent protein and radioactively labeled with Cr-51 were infused via the tail vein in wild-type C57BL/6 mice. After 5 min, 1, 24, or 72 h, mice were sacrificed and blood, lungs, liver, spleen, kidneys, and bone marrow removed. One hour after MSC infusion the majority of Cr-51 was found in the lungs, whereas after 24 h Cr-51 was mainly found in the liver. Tissue cultures demonstrated that viable donor MSC were present in the lungs up to 24 h after infusion, after which they disappeared. No viable MSC were found in the other organs examined at any time. The induction of ischemia-reperfusion injury in the liver did not trigger the migration of viable MSC to the liver. These results demonstrate that MSC are short-lived after i.v. infusion and that viable MSC do not pass the lungs. Cell debris may be transported to the liver. Long term immunomodulatory and regenerative effects of infused MSC must therefore be mediated via other cell types.

## Introduction

Mesenchymal stem cells (MSC) are considered as a potential therapy for a wide variety of degenerating and immunological disorders (Giordano et al., 2007; Reinders et al., 2010; Salem and Thiemermann, 2010). Animal models demonstrate that MSC induce the repair of injured organs and ameliorate inflammatory processes (Morigi et al., 2008; Aurich et al., 2009; Gonzalez-Rey et al., 2009; Fisher-Shoval et al., 2012). The encouraging results in such models have initiated the translation of MSC therapy in clinical trials in a range of disorders, including graft versus host disease, inflammatory bowel disease, and cardiac infarct (Le Blanc et al., 2008; Hare et al., 2009; Duijvestein et al., 2010). Trials in multiple sclerosis (Freedman et al., 2010), systemic lupus erythematosus (Liang et al., 2010), and in organ transplantation (Perico et al., 2011; Popp et al., 2011; Tan et al., 2012) are currently ongoing or in preparation.

A problem with the interpretation of results and optimization of trials with MSC is a lack of understanding of the mechanism of action of MSC. While MSC have the capacity to differentiate into multiple cell types (Pittenger et al., 1999; Long et al., 2005), secrete growth factors that stimulate the proliferation and differentiation of other cells (Lee et al., 2011), and inhibit the proliferation of immune cells *in vitro* via the secretion of anti-inflammatory factors (Di Nicola et al., 2002), it is unknown whether these mechanisms are operational after administration of MSC. Moreover, there is controversy about the localization and persistence of MSC in the body after administration. The route of administration is an important factor determining the fate of MSC. The favorite route of administration in human is intravenously, as this has proven to be safe and allows the administration of large amounts of MSC. Tracking studies have shown that the majority of MSC localize to the lungs after intravenous infusion (Barbash et al., 2003; Kraitchman et al., 2005; Fischer et al., 2009; Assis et al., 2010). The detainment of MSC in the lungs is due to space restriction, as cultured MSC are more than 20 μm in diameter (Crop et al., 2010) and therefore much larger than circulating immune cells and larger than the width of the micro-capillaries of the lungs. Administration of MSC via alternative routes leads to detainment of MSC in other filtering organs. For instance, MSC administered via the portal vein are found in the liver (Shi et al., 2010), while MSC administered in tissues like muscle, spine, and fat pads remain present locally up to several weeks (Boulland et al., 2012; Hu et al., 2012; Nam et al., 2012).

After intravenous administration, MSC tend to disappear from the lungs within hours and migrate to other tissues such as the spleen and liver (Devine et al., 2003; Kraitchman et al., 2005) and preferentially to sites of injury (Chapel et al., 2003; Assis et al., 2010; Jackson et al., 2010; Jin et al., 2012). However, care should be taken with interpreting these results. Studies examining the distribution of MSC after intravenous infusion rely on PCR techniques, immunofluorescence, or bioluminescence to detect DNA, fluorescence label, or luciferase enzyme activity from infused MSC, but do not encompass the detection of living MSC. It is not unlikely that label is detected in dead MSC or in macrophages that have phagocytosed MSC. Detection of label provides therefore no information on the localization and persistence of living MSC. Many studies examining the distribution of MSC use (severely) immuno-compromised recipient animals (Pereira et al., 1995; Liechty et al., 2000; Devine et al., 2003; Boulland et al., 2012). In most human studies, MSC recipients will have a more functional immune system and this is likely to affect the survival of MSC. The idea that MSC may not survive long after administration is supported by evidence demonstrating that the majority of MSC become apoptotic after administration (Liu et al., 2012).

In the present study we examined the localization of living MSC after intravenous infusion in immunocompetent mice by re-establishing cultures of administered MSC. Bone marrow-derived MSC of C57BL/6 mice that constitutively express DsRed were infused via the tail vein of wild-type C57BL/6 mice. After 5 min, 1, 24, or 72 h blood was taken and lung, spleen, liver, kidney, and bone marrow removed, MSC isolated and brought into culture. After 1 week of culture, the presence of adherent DsRed-MSC was analyzed by microscopy and flow cytometry. The outcome was compared to the results obtained from distribution experiments with radioactive labeled MSC in the same model.

## Materials and methods

### Ethics statement

All animal experiments were carried out in accordance with European communities council directive (86/609/EEC) and institutional guidelines for animal care after local ethics committee approval (Ethics committee for animal laboratories, Medical Faculty, University of Regensburg, 93042, Regensburg, Germany). The MSC tracking studies were conducted after approval by the local authorities governing health care (Regierung der Oberpfalz, Emmeransplatz 8, 93047, Regensburg, Germany, www.ropf.de; AZ: 54-2532.1-33/08).

### Experimental animals

We used DsRed C57BL/6 mice (Jax, stock number 006051; http://www.jaxmice.jax.org) as MSC donors. These transgenic mice, which carry an Actb-DsRed.T3 transgene, express the red fluorescent protein variant DsRed.MST under the control of the chicken beta actin promoter coupled with the cytomegalovirus (CMV) immediate early enhancer. All tissues of homozygotes are red fluorescent. 6–8-weeks-old wild-type C57BL/6 mice (Charles River Laboratories, Sulzfeld, Germany) were used as MSC recipients. For some experiments, Rag2^−/−^ × common cytokine receptor γ-chain^−/−^ double knock out (Rag2^−/−^ × γ-chain^−/−^) mice lacking T, B, and NK cells were used as MSC recipients. Laboratory animals were housed with access to food and water provided *ad libitum*. Water was provided via standard lab water bottles which were replenished daily. Cages were cleaned weekly. All veterinary procedures were performed only with sedated animals. All efforts were taken to ameliorate any suffering.

Liver ischemia-reperfusion injury was induced according to Abe et al. (2009). In brief, ischemia-reperfusion injury was induced by placing an atraumatic clip across the portal vein, hepatic artery, and bile duct just above the branching to the right lateral lobe. The median and lateral lobe (approximately 70% of the liver) showed significant blanching. After 45 min of ischemia, the clamp was removed and the liver reperfused.

### Isolation and culture of MSC

MSC were isolated from tibias and femurs of DsRed C57BL/6 mice by flushing. The obtained cell suspension was washed and plated in tissue culture flasks in MEM alpha supplemented with 10% heat-inactivated fetal calf serum (FCS), and 100 U/mL penicillin and 100 mg/mL streptomycin (1% p/s) (all Invitrogen, Karlsruhe, Germany). After 2–3 days non-adherent cells were removed. Plastic adherent cells were removed by trypsinization after reaching 70–80% confluency. After the first passage, CD11b^+^ cells were depleted from the cultures by MACS (Miltenyi, Bergisch Gladbach, Germany). Cells were maintained at 37°C, 5% CO_2_, and 95% humidity, culture medium refreshed twice weekly and used for experiments between passage 2 and 5.

### Characterization of DsRed-MSC

DsRed-MSC were examined for DsRed expression by fluorescence microscopy and flow cytometry using a FACS Calibur (BD Biosciences, San Jose, USA). For surface marker characterization, MSC were harvested and washed twice in ice-cold phosphate-buffered saline (PBS). Stainings were performed in 50 μl of pre-diluted FITC-conjugated anti-mouse CD34, CD11b, Sca-1 or MHC-I, and APC-conjugated anti-mouse CD44, CD11c, or CD117 antibody (all from BD Biosciences, Heidelberg, Germany). After 20 min incubation at 4°C in the dark, 400 μl of PBS was added, and the cells analyzed with the FACS Calibur flow cytometer.

Differentiation of MSC into adipocytes was achieved by plating MSC into six-well plates in expansion medium without FCS for 2 days. Differentiation was induced by culturing the cells in expansion medium without FCS supplemented with insulin (15 U/ml; Sanofi-aventis, Paris, France), dexamethasone (10^−6^ M; Sigma-Aldrich, St. Louis, USA), goat serum (5 ml/100 ml; PromoCell, Heidelberg, Germany), and 3-isobutyl-1-methylxanthin (0.1 mg/ml; Sigma-Aldrich) for 3 days. The cells were then cultured in expansion medium without FCS supplemented with insulin (15 U/ml; Sanovi-aventis) for 5 days. Cell differentiation into adipocytes was confirmed by oil red O staining. Cells were washed in cold PBS, fixed with 10% formaldehyde at 4°C for 10 min, and then incubated with 5 mg/ml oil red O solution (Sigma-Aldrich, Munchen, Germany) for 2 h at room temperature.

To induce osteogenic differentiation, MSC were seeded in six-well plates at a density of 15,000 cells per cm^2^ in DMEM without FCS for 1 day. Cells were then treated with osteogenic medium for two weeks, changing the medium twice a week. Osteogenic medium consisted of DMEM supplemented with 0.1 μM dexamethasone, 0.3 mM ascorbic acid, and 10 mM α-glycerolphosphate (all Sigma-Aldrich). Osteogenic differentiation was assessed by von Kossa staining. Cells were covered with 5% silver nitrate solution for 40 min in bright light followed by an incubation step in UV light for 2 min. After rinsing with distilled water, cells were incubated for 5 min in 1% pyrogallol (Sigma-Aldrich) and rinsed again. Non-specific staining was removed by washing the cells in 5% sodium thiosulfate (Sigma-Aldrich) for 5 min.

For examination of the immunomodulatory capacity of DsRed-MSC, splenocytes of C57Bl/6, or Balb/c mice were labeled with CFSE (Vybrant Cell Tracer Kit, Molecular Probes, Eugene, Oregon, USA) and stimulated with 2 ng/ml Concanavalin A (ConA) in MEM alpha with 10% FCS and 1% p/s. DsRed-MSC were added at a 1:5 ratio. After 3 days, splenocytes were collected, stained for CD3 and CD4 (BD Biosciences), and analyzed on the flow cytometer.

### Infusion of DsRed-MSC

DsRed-MSC were trypsinized and washed twice with PBS. A suspension of 500,000 DsRed-MSC in PBS was infused via the tail vein of C57BL/6 mice or Rag2^−/−^ × γ-chain^−/−^ mice. Control animals received PBS only. Mice were sacrificed after 5 min, 1, 24, or 72 h, blood collected, and lungs, liver, spleen, kidneys, and bone marrow removed.

### Organ harvest and re-isolation of DsRed-MSC

#### Blood

Approximately 1 ml blood was collected and red blood cells lysed in red cell removal buffer (Roche, Germany). The cells were then washed and plated out in tissue culture flasks in MEM alpha with 10% FCS and 1% p/s.

#### Lungs, kidneys, spleen

Organs were minced with a scalpel knife and incubated in 0.5 mg/ml collagenase in PBS for 30 min in a shaker at 37°C. The tissue was then put several times through a 19 G needle with a syringe, washed, taken up in MEM alpha with 10% FCS and 1% p/s, filtered through a 100 μm cell strainer, and plated out in tissue culture flasks.

#### Liver

Livers were harvested and prepared with the Gentle MACS dissociator according to the protocol (Miltenyi, Bergisch Gladbach, Germany) without the final centrifugation step to obtain a single cell suspension including all liver cells. Cells were washed, taken up in MEM alpha with 10% FCS and 1% p/s, and plated out in tissue culture flasks.

#### Bone marrow

Tibias and femurs were flushed with PBS and the cell suspension was washed and plated out in tissue culture flasks in MEM alpha with 10% FCS and 1% p/s.

After 2 and 7 days of culture, non-adherent cells were removed by washing and the adherent cells examined for the presence of DsRed-MSC by fluorescence microscopy.

### Infusion of radio-labeled DsRed-MSC

DsRed-MSC were radio-labeled using a protocol described by Sprent (1976). Briefly, MSC were diluted to a concentration of 20 × 10^6^ /ml and incubated with 20 μCi/ml Cr-51 Sodium (PerkinElmer, Waltham, MA, USA) for 1 h followed by two washes with medium. Subsequently, 500,000 radio-labeled cells were infused via the tail vein of C57BL/6 mice. The animals were sacrificed and blood and organs removed after 1 or 24 h. Organs were place in vials and radioactivity measured as counts/min with a 1470 Wallac Wizard gamma counter (PerkinElmer).

## Results

### Characterization of DsRed-MSC

**Figure 1:**
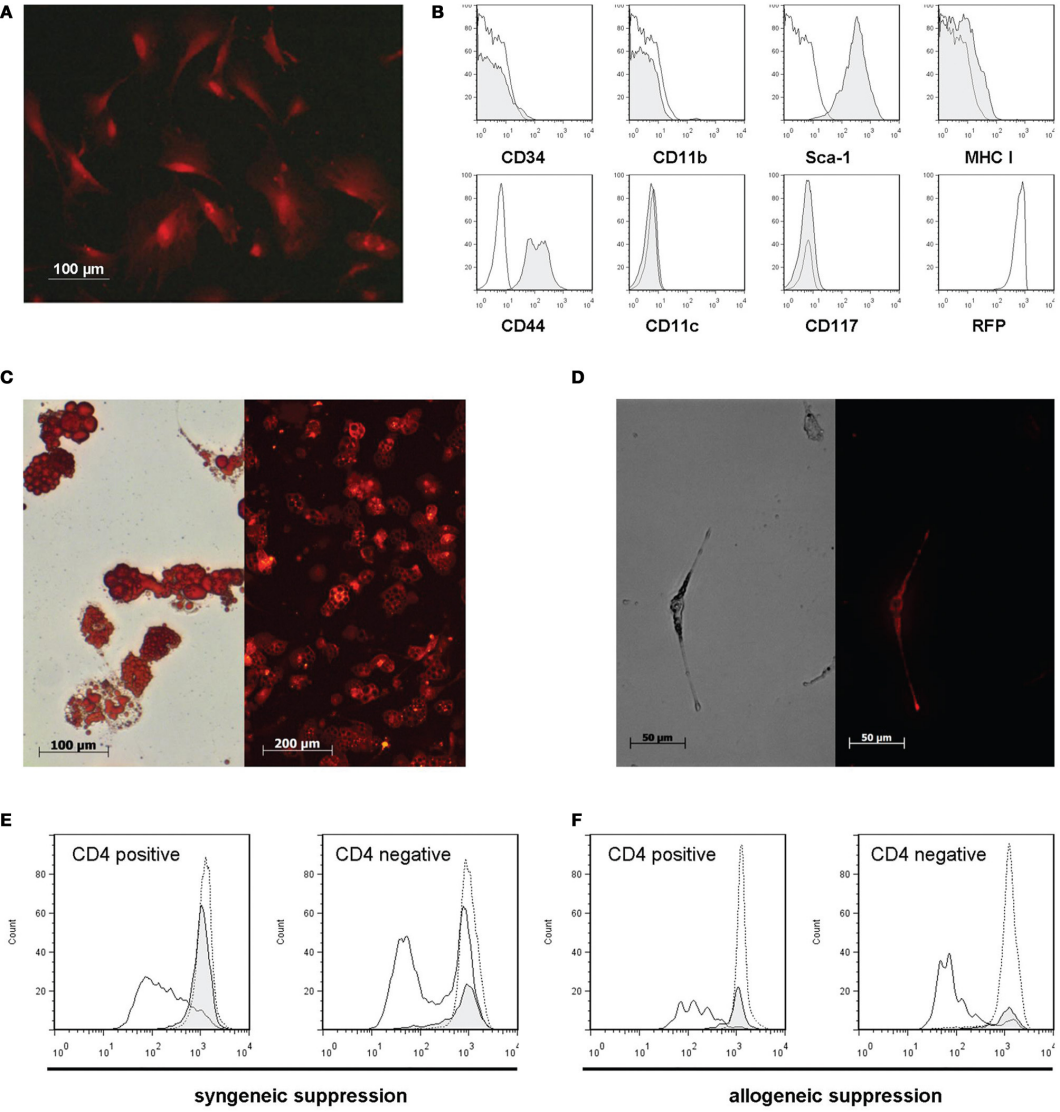
**DsRed-MSC are phenotypically and functionally comparable to wild-type MSC**. **(A)** Fluorescence microscopy of plastic adherent DsRed-MSC in culture shows bright red fluorescence. **(B)** Flow cytometric analysis demonstrates that DsRed-MSC are negative for the expression of CD34, CD11b, CD11c, and CD117, show weak expression of MHC class I, and are positive for Sca-1, CD44, and RFP (DsRed). **(C)** DsRed-MSC are capable of differentiating into adipocytes, as demonstrated by staining of lipid vesicles by Oil-Red-O (left), while remaining red fluorescent (right). **(D)** DsRed-MSC are capable of differentiating into osteoblasts, indicated by positive silver nitrate staining for calcium deposits (left), while remaining red fluorescent (right). (**E** and **F**) DsRed-MSC suppress ConA induced proliferation of CD4 positive and negative T cells efficiently, determined by CFSE dilution on day 3 (solid line: ConA stimulated T cells, gray shaded curve: ConA stimulated T cells + MSC, dotted line: non-stimulated T cells). DsRed-MSC suppressed the proliferation of syngeneic C57BL/6 responder T cells **(E)** as well as allogeneic Balb/c responder T cells **(F)**. Representative data of 3 experiments shown.

DsRed-MSC isolated from the bone marrow had a typical fibroblast-like appearance in culture, similar to wild-type MSC (Figure 1A). They expressed the MSC markers Sca-1 and CD44, and were negative for CD34, CD11b, CD11c, and CD117. They had only a weak expression of MHC class I, and constitutively expressed high levels of DsRed (Figure 1B).

To show that DsRed-MSC are able to differentiate like wild-type MSC, we cultivated them under adipogenic and osteogenic conditions. After 2 weeks in culture, DsRed-MSC started to differentiate into adipocytes, as demonstrated by positive oil red O staining of lipid-filled vesicles (Figure 1C, left). Importantly, these differentiated cells still remained red fluorescent (Figure 1C, right). After 2 weeks in osteogenic differentiation medium, DsRed-MSC started to deposit calcified nodules, which stained black with silver nitrate (Figure 1D, left). Also, the differentiated osteoblasts remained red fluorescent (Figure 1D, right).

DsRed-MSC furthermore shared the property of wild-type MSC of inhibiting the proliferation of ConA stimulated splenocyte proliferation. The proliferation of CD3^+^CD4^+^ T cells and CD3^+^CD4^−^ T cell subsets was significantly inhibited on day 3 by DsRed-MSC added at a 1:5 ratio. DsRed-MSC inhibited both syngeneic (C57BL/6 Responders cells) (Figure 1E) as well as allogeneic responder cells (Balb/c Responder cells) (Figure 1F).

### Distribution of radioactive labeled syngeneic MSC

**Figure 2:**
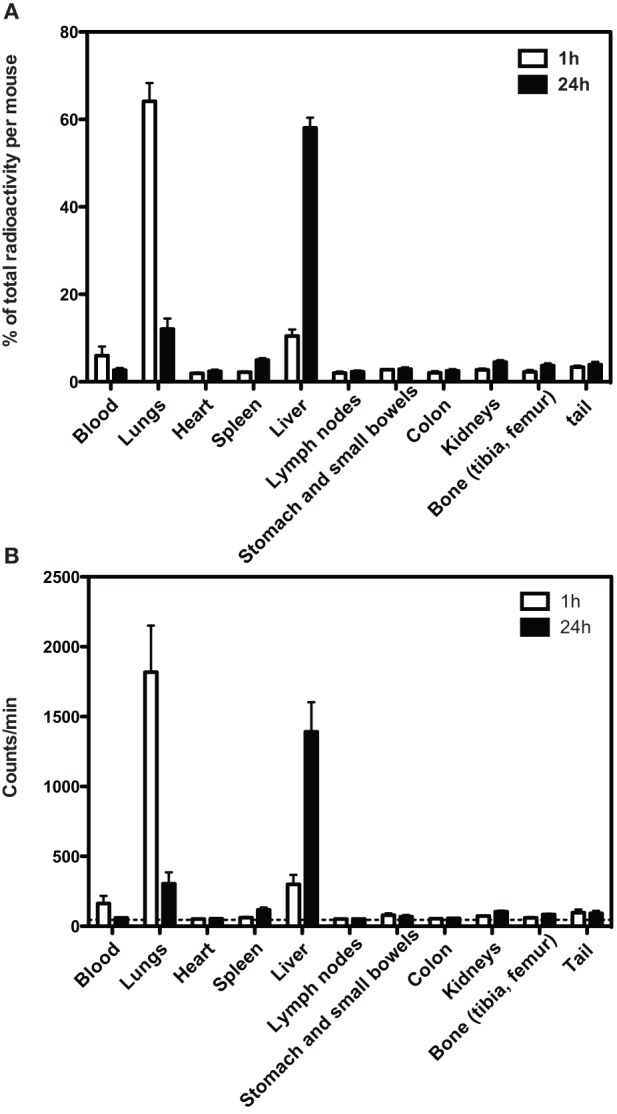
**Distribution of Cr-51 radioactivity after infusion of Cr-51 labeled DsRed-MSC**. **(A)** One hour after intravenous injection, more than 60% of total radioactivity is located in the lungs and approximately 10% in the liver. Other tissues contain only residual radioactivity. After 24 h, the majority of radioactivity is located in the liver. **(B)** As in **(A)**, but absolute counts shown. Dashed line indicates background radioactivity. Cervical, axillary, inguinal, hepatic, para-aortal, and mesenteric lymph nodes were collected. Average data of 2 experiments is shown.

We radio-labeled DsRed-MSC and followed their distribution after intravenous infusion by tracking the radioactive signal. One hour after administration of MSC, the majority (60%) of radioactivity was found in the lungs, while a smaller proportion was found in the liver (Figure 2). After 24 h, the amount of radioactivity in the lungs was strongly reduced, while the amount of radioactivity in the liver was increased. A small amount of radioactivity was found in the spleen. Radioactivity in other organs was around background levels.

### Presence of living DsRed-MSC in tissue cultures

To examine whether infused MSC home to lungs, liver and perhaps other tissues as living cells, DsRed-MSC (500,000) were infused via the tail vein in syngeneic C57BL/6 mice and tissues isolated at various time points after infusion and brought into culture to detect the presence of viable DsRed-MSC. Thus, after 5 min, 1, 24, or 72 h mice were sacrificed and blood, lungs, liver, spleen, kidneys, and bone marrow collected and cell cultures established in MSC-supporting culture medium. Adherent cell cultures reaching confluency after 7–10 days were obtained from lung, spleen, kidney, and bone marrow tissues. The cultures consisted of multiple cell types, including macrophage-like cells, endothelial cells, and fibroblastic cells. Single colonies of fibroblastic cells were obtained from blood and liver tissue.

**Figure 3:**
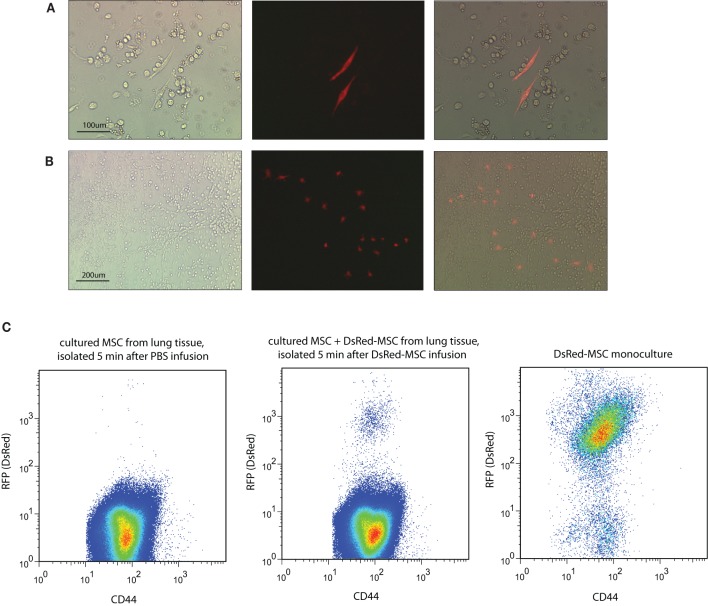
**Viable MSC are detected in lung tissue only after intravenous infusion. (A)** Brightfield, immunofluorescent, and merged microscopic images show two DsRed-MSC in a two days-old culture of lung tissue established 5 min after MSC infusion. **(B)** After 7 days of culture clusters of DsRed-MSC are seen. **(C)** Flow cytometric analysis of cultures established from lung tissue 5 min after DsRed-MSC infusion cultured for 7 days revealed a distinct population of CD44^+^ DsRed-MSC (middle plot), while in cultures of PBS treated animals no DsRed-MSC were present (left plot). Mono-cultures of DsRed-MSC served as gating control (right plot). Five animals were included per group. Representative data shown.

Analysis by fluorescence microscopy revealed that cultures obtained from blood collected 5 min after MSC infusion contained no DsRed-MSC. However, lung tissue cultures contained red fluorescent cells, indicating that living donor MSC were present in the lungs 5 min after infusion of DsRed-MSC. Interestingly, after 2 days of culturing multiple DsRed-MSC were frequently shown in close proximity, suggesting that the cells were proliferating (Figure 3A). After 7 days of culture, DsRed-MSC were mostly found in colonies rather than equally distributed throughout the cultures (Figure 3B). Flow cytometric analysis of lung tissue after 7 days of culture confirmed the presence of DsRed-MSC expressing the MSC marker CD44^+^ (Figure 3C). Cultures from spleen, kidney, liver, and bone marrow established 5 min after MSC infusion contained no DsRed-MSC.

DsRed-MSC were also present in lung cultures established 1 h after MSC infusion, but their numbers were strongly reduced in cultures obtained at 24 h. No DsRed-MSC were detected in lung cultures established 72 h after MSC infusion, suggesting that the infused MSC were either no longer viable or had migrated to other tissues. Surprisingly, living DsRed-MSC were not detected in cultures of any of the other tissues established at 1, 24, or 72 h after MSC infusion (Table 1).

**Table 1:** **The presence of living MSC in tissues 5 min, 1, 24, and 72 h after infusion of 500,000 DsRed-MSC via the tail vein at *T* = 0**.

	***T* = 0**	***T* = 5 min**	***T* = 1 h**	***T* = 24 h**	*T* = 24 h liver IRI^*^	***T* = 72 h**
Blood	+	−	−	−	−	−
Lung	−	+	+	+	+	−
Liver	−	−	−	−	−	−
Spleen	−	−	−	−	−	−
Kidney	−	−	−	−	−	−
Bone marrow	−	−	−	−	−	−

Five animals were included per group.

* Liver IRI: 45 min of ischemia-reperfusion injury of the liver, MSC infusion 1 h before ischemia-reperfusion injury.

### Presence of living DsRed-MSC in ischemia-reperfusion injury liver tissue

To examine whether organ injury would provide a trigger for MSC to migrate to the organ, ischemia-reperfusion injury was induced in the liver of mice by clamping the hepatic artery, portal vein, and bile duct for 45 min in order to prevent about 70% of the liver lobes from blood supply. One hour before ischemia-reperfusion injury, 500,000 DsRed-MSC were infused via the tail vein. At this time point we know from the previously described experiments that living MSC are present in the lung. Twenty-four hours after reperfusion, the organs were removed and MSC brought into culture. No living DsRed-MSC were detected in liver tissue after ischemia-reperfusion injury (Table 1). DsRed-MSC were present in lung tissue, like in control animals.

### Presence of living DsRed-MSC in Rag2^−/−^ × γ-chain^−/−^ recipients

To determine whether NK, T, and B cells were responsible for the rapid disappearance of living DsRed-MSC after infusion, DsRed-MSC were infused in Rag2^−/−^ × γ-chain^−/−^ double knock-out mice that are deficient for these cells, and organs removed and cultures established. Like in wild-type mice, living DsRed-MSC were found in lung tissue up to 24 h after infusion, but not in any other tissue at any time point (results not shown).

## Discussion

MSC therapy has shown to be effective as an immunomodulatory and regenerative therapy in a number of animal models, including transplant models (Popp et al., 2008), experimental colitis (Gonzalez et al., 2009), pancreatitis (Jung et al., 2011), experimental multiple sclerosis (Fisher-Shoval et al., 2012), and several others. The mechanisms that mediate the effects of MSC in these models are not clear. The old dogma that administered MSC engraft and differentiate in specialized cell types has been abandoned, whereas the proposition that the effects of MSC are mediated via the secretion of trophic and immunoregulatory factors has gained in popularity.

In the present study we demonstrated that MSC accumulate in the lungs within the first few hours after intravenous infusion. This is in agreement with earlier findings (Barbash et al., 2003; Kraitchman et al., 2005; Assis et al., 2010). Importantly, we were able to demonstrate that at least some of the exogenous MSC remained viable in the lungs up to 24 h after infusion. Re-culturing of these MSC demonstrated that they maintained their proliferation capacity. During the first 24 h after infusion, living MSC were not found in blood, liver, spleen, kidney, or bone marrow. After 24 h, living MSC disappeared from the lungs, but did not reappear in the other tissues examined, suggesting they did not survive long term in the recipient animals.

As it has been suggested that MSC migrate to sites of injury, we induced ischemia-reperfusion injury in the liver and examined whether viable administered MSC would appear in the injured liver. We found, however, no living donor MSC in the injured liver, indicating there is no difference in the migration of viable MSC to injured and non-injured organs.

The identification of viable MSC after infusion has not been demonstrated earlier and shines a new light on the fate of MSC after administration. Other studies reported the migration of MSC to various sites, particularly liver and spleen, and to sites of injury. In our experiments, radioactivity was found in the liver and spleen 24 h after injection of radio-labeled MSC but we failed to isolate viable MSC from these organs. Our data indicate that living MSC do not pass the capillary bed of the lungs after intravenous infusion. It is therefore likely that previous studies describing MSC in other tissues detected MSC-label (e.g., radioactivity, fluorescence) from MSC debris or from phagocytosed MSC rather than living MSC. Our studies suggest that other routes of administration have to be investigated if MSC need to be delivered to tissues other than the lungs. For instance, administration via the portal vein could be used for delivery of MSC to the liver, while arterial administration may deliver MSC to specific organs. The survival of MSC administered via such alternative routes could be examined by the methods used in the present paper.

How intravenously administered MSC disappear from the lungs is not clear at this stage. One possibility could be that MSC are damaged by shear forces after infusion. However, the survival of MSC for up to 24 h in the lungs suggests that their removal is caused by other mechanisms. Immune cells may well be involved in this process. In the present study, the administered MSC were of syngeneic origin, which would suggest a role for cells of the innate immune system in the removal of MSC. Activated NK cells have been shown to be capable of lysing autologous MSC *in vitro* (Spaggiari et al., 2006; Crop et al., 2011). We, however, demonstrated that infused MSC do not have an increased life span in mice that lack NK cells. Other cells of the innate immune system, in particular macrophages, may play a more important role. If the innate immune system is responsible for the loss of administered MSC, it is questionable whether conventional immunosuppressive drugs would be capable of preventing the removal of MSC, as these drugs mainly target the adaptive immune system.

Our data clearly demonstrate the short-term survival of infused MSC and a lack of distribution of viable MSC beyond the lungs. Nevertheless, several studies have demonstrated beneficial effects of MSC in a variety of disease models (Gonzalez et al., 2009; Semedo et al., 2009; Kanazawa et al., 2011) even when MSC were no longer around (Yang et al., 2012). The question now arises how these effects are mediated. It seems clear that delivery of MSC to a site of injury is not required for a therapeutic effect. It has been hypothesized that apoptosis of infused cells can trigger an immunomodulatory response (Thum et al., 2005) and recently it was demonstrated that macrophages adapt an immunoregulatory function after phagocytosis of dead (MSC) (Lu et al., 2012). Our results suggest this process may happen in the lungs and from there develop into a response that eventually targets the immune response at sites of inflammation and injury. Future research will have to reveal which signals conduct this response through the body.

### Conflict of interest statement

The authors declare that the research was conducted in the absence of any commercial or financial relationships that could be construed as a potential conflict of interest.
